# Manuring practices in the first millennium AD in southern Sweden inferred from isotopic analysis of crop remains

**DOI:** 10.1371/journal.pone.0215578

**Published:** 2019-04-18

**Authors:** Mikael Larsson, Jakob Bergman, Per Lagerås

**Affiliations:** 1 Department of Archaeology and Ancient History, Lund University, Lund, Sweden; 2 Department of Statistics, Lund University, Lund, Sweden; 3 The Archaeologists, National Historical Museums, Lund, Sweden; University at Buffalo - The State University of New York, UNITED STATES

## Abstract

This study uses crop stable nitrogen isotope analysis of charred grain to explore manuring practices in arable production at the affluent regional center Uppåkra and a set of smaller surrounding sites, dating to the first millennium AD in southern Sweden. The isotopic analysis focuses on hulled barley, the principle crop in the Scandinavian Iron Age, and the minor crops: bread wheat, emmer wheat, rye and oat, are included to compare manuring practices in cultivation of other crop species during this period. A field experiment was first conducted to establish relationships between manuring and δ^15^N values in modern grain from known growing conditions. The data formed an interpretive framework to reconstruct past agricultural practices and manuring intensity in the archaeological study area. Our results from the ancient grains have demonstrated that barley from the early phase in the study area (AD 0–200) varies widely in its δ^15^N values, reflecting mixed manuring regimes. In the following periods (AD 200–1000), isotopic values are relatively high overall, indicating systematic input of manure. In this paper, we explore whether the isotopic data that indicates sustained and high manuring levels could reflect the wealth of Uppåkra and its surrounding areas by showing prosperity also in its agricultural production, since intensive manuring would have required more resource and labor investments. The new crop nitrogen isotopic data shed light on the agricultural practices of a long-lived Iron Age center and its surrounding areas.

## Introduction

During the Scandinavian Iron Age (500 BC–AD 1050), several large and affluent settlements developed which occupied central positions of economic and political power [1,2]. One of these settlements was the regional center Uppåkra in southern Sweden, dating back to the first century BC, and it remained an important site for over a millennium [3–6]. Such continuous habitation of a settlement was rare in northern Europe at that time.

The longevity of the regional center Uppåkra, its large settlement area (40 ha) and the fact that it was densely inhabited raises questions about its food supplies and production of staple grain. Depending on the fertility of the soil, cereal crops can be grown in the same field for years in succession, but continuous cultivation will likely cause nutrient depletion in soils, and adversely affect soil quality and reduce crop yield [7]. Replenishment of nutrients following their loss from the soil could involve fertilization aimed to maintain and enhance crop yield. Farmers could improve soil condition by application of manures, such as domestic waste and farmyard animal manure [8,9], or clear-cutting of forest by burning [10]. Manures applied to the soils could increase the organic matter content and the microbiological activity, as well as improve soil structure [11]. At present, our knowledge of prehistoric manuring practices in Sweden are elusive and are founded mainly on circumstantial evidence of seeds from nitrogen demanding weed species found among charred cereal grain from archaeological sites [12].

To address questions relating to ancient farming practices, several isotopic studies of archaeobotanical remains have presented evidence that is consistent with the practice of manuring [13–17]. The analysis of N isotopic data in plants has become a method to investigate the growing conditions of plants. Many environmental and cultural variables may influence the N isotopic compositions of plant-soil systems, namely inputs, outputs and nitrogen cycling process [18]. Any process that leads to nitrogen losses in soils translates into a more enriched δ^15^N value of the remaining available soil, and this is reflected in plant δ^15^N values [19]. Natural environmental factors that have been found to influence the δ^15^N values of plants include climate, water stress, salinity and soil nutrient cycling processes [20–26]. With respect to agricultural systems, a major influence on crop δ^15^N is the input of ^15^N rich manure [27]. Plants growing in soils with higher δ^15^N nitrogen inputs, e.g. from manure, can be expected to have greater δ^15^N values [27,28]. Manured soil contains relatively more of the heavier isotope ^15^N than non-manured soil as the lighter isotope of nitrogen, ^14^N, volatilizes easily, giving the manured soil a higher δ^15^N value [10,29]. Manured crops tend therefore to have higher δ^15^N values because the manure has a higher δ^15^N value than the N pool in the non-manured soil. Burning of vegetation or shifting cultivation will also have the effect of increasing plant δ^15^N value [10]. Given the variable factors that can influence N isotopic compositions in plants, and the complexities of N isotopic biogeochemistry in plant-soil systems, it is not possible to directly link δ^15^N measurements of ancient plants to manuring. Nonetheless, numerous studies in archaeology have demonstrated higher δ^15^N values for plants fertilized with animal manures relative to unfertilized plants [28,30,31]. Measuring δ^15^N values of ancient charred grains thus has the potential to shed light on agricultural practices in the past, with respect to the nutrient status of ancient crops to identify manuring intensity.

**Fig 1:**
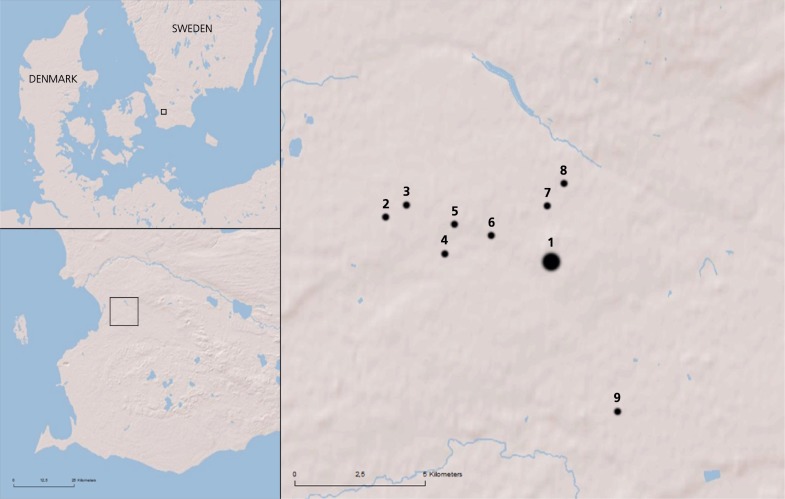
Location of the study area in southern Sweden. Sites providing archaeobotanical samples for stable isotope analysis: (1) regional center Uppåkra, (2) Hjärup 21:36, (3) Hjärup 7:1, (4) Hjärup 9:8, (5) Åttevägen, (6) Uppåkra 12:110, (7) Uppåkra 2:14, (8) Uppåkra 2:25, (9) Stanstorp 5:1.

This study uses crop stable nitrogen isotope analysis to explore manuring intensity in arable production at the regional center Uppåkra and a set of surrounding sites during the first millennium AD (Fig 1). The smaller settlements, characterized as farmsteads, are located within 6 km of the main site and these are, in contrast to the regional center, rather short-lived but together they represent the nearby hinterland for the first millennium AD.

The study was performed in two steps. Step one was a field experiment on modern crops to establish relationships between manuring and δ^15^N values in grain. The aim was to assess isotopic values in grain for within- and between-plant variation, and to measure the effect of known levels of farmyard manure to facilitate interpretation of isotopic values of archaeological grain from sites in the study area. Step two was a study of δ^15^N values in ancient grain of charred hulled barley and of the minor crops: bread wheat, emmer wheat, rye and oat from nine archaeological sites in the study area: the regional center Uppåkra and eight smaller surrounding sites. The aim was to determine: 1) the manuring intensity within its farming practices and whether the application of manure varied between cereal species, 2) whether manuring practices varied between sites and across time within the study area, and 3) whether variation in δ^15^N values in individual sample contexts indicate grains gathered from a single location or multiple locations.

Grain from the field experiment has been grown in the same geographical area as the archaeological sites with similar geological and environmental conditions. Soil types in this area consist of uniform Quaternary deposits, which are dominated by clay till and overlying chalk bedrock [32] and the area has a maritime climate with a relatively narrow annual temperature range [33].

This paper builds on the nitrogen isotopic analysis approaches for studying past manuring practices [28,30,34–36] by exploring if the affluence at the regional center Uppåkra is also reflected in its agricultural production. If intensive cultivation was required to meet the demand of staple grain at the regional center, this may also have resulted in a greater need for intensive manuring on arable lands. We envisage that this would have required more investment, with respect to availability of manure and ownership of animals, and can therefore reflect an aspect of prosperity in cereal cultivation. Studying a confined geographical area across a millennium, we explore if sustained use of manure was the basis for its long-term arable production and if cultivation practices using manure varied between sites and cereal species. These are the first Iron Age sites in Sweden from which charred grain have been isotopically analyzed.

## Materials and methods

### Isotope analysis of cereal grain

A single-grain analytical method of δ^15^N was undertaken for both the archaeological and modern grains in this study. The analytical method allows the variance in δ^15^N of individual samples to be calculated, but also individual isotopic measures of grains from a sampled population can be pooled to calculate the sample mean. This approach was carried out to study the variability of isotopic values in grains within individual samples and between sampled contexts.

447 grains were subjected to isotopic analysis: 400 carbonized grains from archaeological samples, and 47 modern non-carbonized grains from the field experiment. Additionally, 87 random duplicate samples were analyzed. Duplicates from multiple aliquots of the same sample were performed to give a sense of the precision associated with the IRMS measurements. (Duplicate data is marked gray in S1 and S2 Tables). Preparation of samples included gentle removal of any visible surface contaminants, such as adhering sediments or plant roots. All grains were documented by measuring dimension (L, B, W) prior to isotopic analysis. The δ^15^N values in charred archaeobotanical grain were corrected for the effect of charring by subtracting 0.3 ‰ [37].

The stable nitrogen isotopic composition of modern and ancient grain samples was determined using a Sercon model 20–20, Continuous Flow Isotope Ratio Mass Spectrometer (CF/IRMS) linked to a Thermo model EA1110 Elemental Analyser (EA) utilising a Carbosieve G separation column. Individual single seed samples were ground to a fine powder in a micro agate mortar and pestle. All isotope standard reference materials (SRMs), in-house quality control (QC) check samples and unknown samples were weighed to 6 decimal places of a gram using a Mettler UMX5 microbalance.

The IRMS calibration for δ^15^N was scale normalized using isotope SRM’s USGS40 (δ^15^N -4.5 ‰ AIR) and USGS41a (δ^15^N +47.6 ‰ AIR) as the lower and upper scale anchors (two point calibration). Sample sequences of 10 unknown samples were bracketed by a low/high SRM pair. Measurement uncertainty for δ^15^N and %N was monitored using 2 well-characterised in-house QC check samples GA1 QC (Glutamic Acid, δ^15^N -5.2 ‰ AIR, 9.5%N) and GA2 QC (Glutamic Acid, δ^15^N -3.1 ‰ AIR, 9.5%N) in every 10 sample sequence. Approximately 10% of the unknown samples were duplicated in each sample sequence.

SRM’s, QC check samples and unknown samples for δ^15^N analysis were weighed according to their elemental composition to ensure that the absolute weights of nitrogen were within the linear response range of the IRMS. Percentage nitrogen values of the samples and QC check samples were determined from the IRMS total beam values referenced to the theoretical nitrogen percentage composition of the SRM’s.

### Field experiment

The field experiment was pursued with two objectives: 1) to examine the difference in grain δ^15^N between plots receiving farmyard manure vs no manure, and 2) to explore within- and between-plant variation in grain δ^15^N. The field experiment was conducted in collaboration with the Swedish University of Agricultural Science (SLU) at Borgeby, 10 kilometers northwest of the archaeological study area. SLU has managed long-term agricultural experiments of manured and unmanured plots (each plot measuring 112.5 m^2^) at the location since 1957 with a four-year rotation system of the same plant species (hulled barley—fodder [mostly of grasses]—winter-sown bread wheat—sugar beets). Application of solid farmyard manure in the experiments shifted in 2007 to farm slurry (liquid manure) of cattle, and spread on the plots using a manure spreader. In the experiments, only manure from certified organic agriculture was used. Manuring rates quantifiable as 25 tonnes per hectare have since the start of the experiments been applied every fourth year, with the most recent addition in 2015. The plots have been ploughed yearly in the autumn and any ground vegetation (e.g., remaining plant straw or weed plants) has been mixed into the soil.

For this study, samples were collected in August 2017 when two-row hulled barley (*Hordeum vulgare* ssp. *distichon*) was cultivated in the rotation cycle. Two experimental plots were chosen: Plot 1. A0 received manure and plot 2. A0 received no manure. The latter plot has not received any nutritional input since the start of agricultural experiments in 1957.

Samples for isotopic analysis were collected by taking four ears from four plants from both plots, in total 8 ears (samples). From each ear, every second grain on one side was sampled for analysis, from the basal to the terminal spikelet. Sample size varied from ear to ear, as the total number of grains on individual ears varied. From ears having fewer grains, every second grain was taken from both sides of the central axis.

### Archaeobotanical materials and sample selection

Archaeobotanical cereal grain collected and analyzed from several archaeological contexts at the regional center Uppåkra and eight surrounding sites during 2001–2017 were used for isotopic analysis (Table 1). The analysis focused on hulled barley (*Hordeum vulgare* ssp. *vulgare*), in part due to the dominance of this cereal type in the archaeobotanical record in the study area, and in part, due to its role as the principal crop during the entire Scandinavian Iron Age [38]. Isotopic analysis of the minor crops, bread wheat (*Triticum aestivum*), emmer wheat (*Triticum dicoccum*), rye (*Secale cereale*) and oat (*Avena sativa*), was used to complement and explore manuring practices of other crop species.

**Table 1:** Chronology of the Swedish Iron Age in the first millennium AD and archaeological sites in this study. ERIA = Early Roman Iron Age; LRIA = Late Roman Iron Age; MP = Migration Period; VP = Vendel Period; VIK = Viking Period.

*Period*	ERIA	LRIA	MP	VP	VIK
*Chronology*	0–200	200–400	400–550	550–800	800–1050
*Archaeological sites*					
**Main site**					
Regional center Uppåkra	√	√	√	√	√
**Surrounding sites**					
Åttevägen	√				
Uppåkra 12:110	√				
Uppåkra 2:14	√				
Uppåkra 2:25	√	√			
Stanstorp 5:1			√		
Hjärup 7:1			√		
Hjärup 21:26				√	
Hjärup 9:8					√

Samples of grain for isotopic analysis were primarily selected from archaeological contexts in which the archaeobotanical material has been interpreted to have formed from single depositional events, this includes layers of charcoal/ash-rich floor deposits formed after a house-fire, overlaying floor layers constructed from clay. However, to increase isotopic data from the study area, other features containing sufficient grain content, such as refuse pits and ovens were included. It is unclear if these latter features represent contexts from one seasonal use, or those used over multiple years.

40 sample contexts were selected for isotopic analysis, 21 from the regional center Uppåkra and 19 combined from the surrounding sites (see S2 Table for sample details). Sample selection for barley covered all sites and periods in the study area. Other cereals, bread wheat, emmer wheat, rye and oat, were less abundant and are restricted to the Early Roman Iron Age and Migration Period. The samples can be divided into five periods: Early Roman Iron Age (n = 10), Late Roman Iron Age (n = 4), Migration Period (n = 18), Vendel Period (n = 4) and Viking Period (n = 4).

Each sample consisted of 10 grains, and separate isotopic measurements were carried out on all individual grains. Sample size was based on previous studies that have concluded that measuring isotopic values from up to 10 individual grains deriving from one archaeological sample covers the full range of variability necessary for calculating sample mean [35,37,39,40]. Charred grains showing well-preserved physical characteristics (Preservation scale 1, 2 or 3, distortion scale 1 or 2) associated with optimal carbonization were selected to minimize isotopic offsets from poorly preserved grains charred in high temperatures [41].

The archaeobotanical samples or their contexts have previously been ^14^C dated. Dates were obtained via literature: sample contexts at regional center Uppåkra [42–45]; from sites in the surrounding area, dates were obtained from archaeological reports [46–51].

All archaeological specimens used in this study (i.e., 400 charred cereal grains) were obtained from the Department of Archaeology and Ancient History, Lund University (address Helgonavägen 3, 223 63 Lund, Sweden, for contact: Dr M Larsson) and from The Archaeologists, National Historical Museums (address Odlarevägen 5, 226 60 Lund, Sweden, for contact: Dr P Lagerås). Collection of plant material (i.e., 8 ears of barley) from the field experiment was done in collaboration with the Swedish University of Agricultural Science (address Ulls väg 16, 75007 Uppsala, Sweden, for contact: Dr G Bergkvist). No permits were required for the described study. For research purposes, access to the remaining archaeological plant material from archaeobotanical assemblages investigated are available at the above institutions for researchers to study upon request.

### Statistical analysis

A two-way analysis of variance (ANOVA) was used to test whether the mean δ^15^N values vary over time and between the regional center Uppåkra and the surrounding sites. As the residual analysis indicated unequal variances between the different time periods, a modified Wilcox test [52] was used instead. Significant differences were further analyzed using pairwise t-tests with the Welch modification to adjust for unequal variances and Bonferroni correction to control for multiple comparisons [53,54].

The variances in δ^15^N were calculated for each sampled archaeological context. These were compared to the variance in δ^15^N in the field experiment plot that received manure (plot 1. A0, see above) using the classic F-test. Significantly larger variances in the sampled archaeological context could indicate grains gathered from multiple locations. The F-test assumes that the measurements are normally distributed. To assess whether normality was reasonable to assume, Shapiro-Wilk’s test of normality [55,56] was performed prior to the F-test. In case the Shapiro-Wilk’s test was significant, the modified Levene test for equality of variance was utilized instead, as this is fairly robust against departures from normality [54]. All calculations were performed using R 3.4.1 [57].

## Results

### Nitrogen isotope results from field experiment

**Fig 2:**
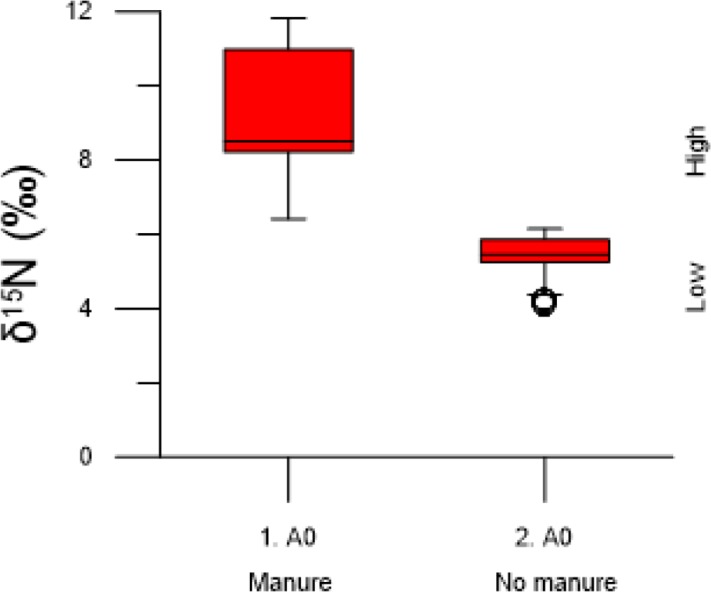
Stable nitrogen isotope values in barley grain from field plots. Each box-whisker plot shows the combined values of the individual grain from all four ears in respective field plots. Plot 1. A0 received manure, and plot 2. A0, received no treatment (no nutritional input since 1957).

The isotopic analysis showed that application of manure to the soil resulted in higher δ^15^N values in grain (Fig 2). The effect of manure was clear between plot 1. A0 receiving manure compared to plot 2. A0 receiving no treatment. Beside isotopic data, the number of plants and grain development on individual ears was observed to be much lower in the non-manured plot compared to the manured plot.

**Fig 3:**
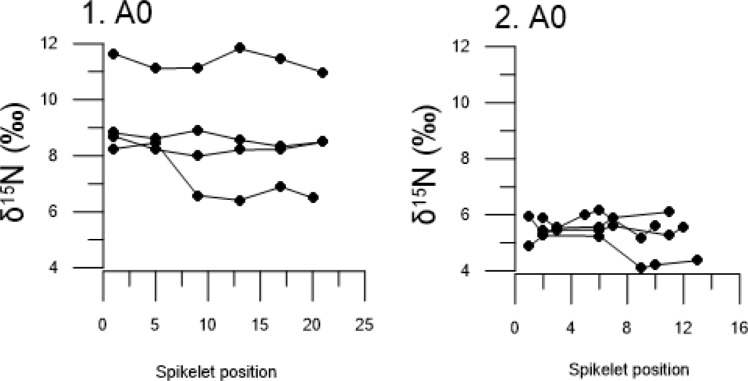
Stable nitrogen isotope values in barley grain from four individual ears for each field plots. Each lined scatter plot in respective field plot represents measurements from individual ears.

Within- and between-plant variation in grain δ^15^N is summarized in Table 2, and individual values are presented in S1 Table. δ^15^N values between grains within a single ear (ear range) showed consistently low levels of variation in both plots. Lowest variation within a single ear was found in non-manured plot 2. A0 at 0.3 ‰ and highest variation was found in plot 1. A0 treated with manure at 2.1 ‰. Average within-plant variation for all 8 ears was 0.9 ‰. Variation between individual ears from different cereal plants within a single plot (plot range) was more noticeable. The experiment showed greater δ^15^N variability between plants in the manured plot compared to the non-manured plot (Fig 3). Plot range was lowest in plot 2. A0 (2.6 ‰) and highest in plot 1. A0 (5.4 ‰).

**Table 2:** Summary of results from isotopic analysis from the field experiment.

			δ^15^N (‰)							
Plot	Ear	No. of analyzed grains	Ear range (min-max)	Mean	Variance	SD	Plot range (min-max)	Mean	Variance	SD
1. A0	1	6	0.7 (8.0–8.7)	8.31	0.06	0.25	5.4 (6.4–11.8)	8.87	2.69	1.64
	2	6	0.9 (11.0–11.9)	11.36	0.11	0.34				
	3	6	0.6 (8.3–8.9)	8.62	0.05	0.21				
	4	6	2.1 (6.4–8.5)	7.18	0.85	0.92				
2. A0	1	6	1.0 (5.2–6.2)	5.70	0.16	0.40	2.1 (4.1–6.2)	5.39	0.32	0.56
	2	5	0.6 (5.5–6.1)	5.79	0.06	0.24				
	3	6	0.3 (5.3–5.6)	5.46	0.01	0.12				
	4	6	1.2 (4.1–5.3)	4.68	0.27	0.52				

### Application of field experiment to manuring intensity in archaeological grain

The isotopic grain measurements from the field experiment provide a framework for interpretations of manuring intensity in archaeological grain. The measured plot range in grain δ^15^N was 6.4–11.8 ‰ in the plot receiving manure (1. A0) with a mean of 8.9 ‰, and 4.1–6.2 ‰ in the plot receiving no manure (2. A0) with a mean of 5.4 ‰.

A cut-off value should be in the interval 6.2–6.4 ‰. We calculate the cut-off value as the weighted mean value of the two samples, with each observation weighted with the inverse of the respective sample standard deviations, which yields a cut-off value of 6.3 ‰. The value is set as a baseline to distinguish between plots receiving low and high inputs of manure. This establishment of a cut-off value rests on the assumption that the soils in which the modern and ancient crops grew are similar before manure addition.

For this study, the baseline is used for interpreting manure intensity from archaeological grain in the study area. Although the baseline is empirically derived, δ^15^N values of the modern crops presented in this study should be interpreted cautiously with respect to past manuring practices. It cannot be assumed that modern N sources used in the experiment (manure slurry from cattle) are directly analogous to those used in the past [26]. Furthermore, application of manure slurry allows for a more even distribution of the manure in the agricultural experiment than application of solid manures in the past. Distribution and mixing of manures into the arable fields in the past may further have been affected by different methods of soil preparations, e.g. ard or scratch-ploughed, which were common in the Scandinavian Iron Age [38,58], compared to deeper-ploughing in the experimental plots. Nevertheless, the results from the experiment to investigate past trends of manure input in the study area are likely broadly applicable.

Given the potential of nitrogen stable isotopes in archaeobotanical research for reconstructing past manuring practices of cereals, the method is not without challenges. Although experimental charring of plant material has demonstrated that this process only slightly alters the δ^15^N values in grain [34,35,37,59], it is less clear if post-depositional processes may alter these values, if at all [19]. Without a high degree of confidence to assess whether the N isotopic compositions derived from archaeobotanical materials are in fact endogenous and not altered in the burial environment, it is necessary to assess the isotopic data cautiously. This is particularly the case when interpretations rest on the difference of a few ‰ in δ^15^N. Comparatively, isotopic analysis on zooarchaeological material can use collagen extracted to display a C:N atomic ratio within an accepted range (minimum %C and %N, collagen yield) [60]. Indication of potential collagen diagenesis or contamination can be used to discard data unlikely to be representative of endogenous isotopic composition [61]. A similar method of assessing preservation does not yet exist for charred plant material. The effect of burial conditions on grain have been investigated in some studies [34,59], but further isotopic research on archaeobotanical material and detailed geoarchaeological analysis of contemporary paleosols is needed to explore the method and to develop a more reliable set of quality indicators.

### Nitrogen isotope results from archaeological grain

**Fig 4:**
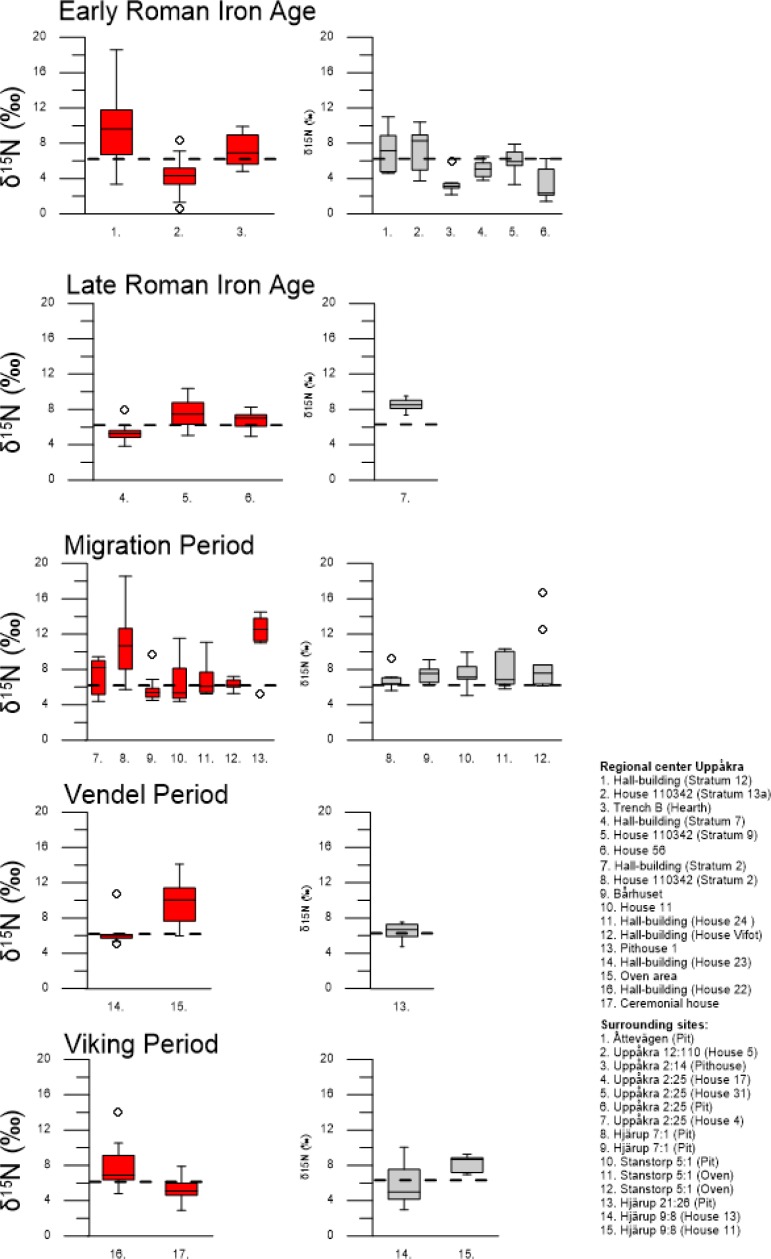
Stable nitrogen isotope values in barley grain from the archaeological study area. Regional center Uppåkra (red) and from the surrounding sites (gray) from respective archaeological periods. Dashed horizontal line at 6.3 ‰ indicates difference between low and high inputs of manure.

**Fig 5:**
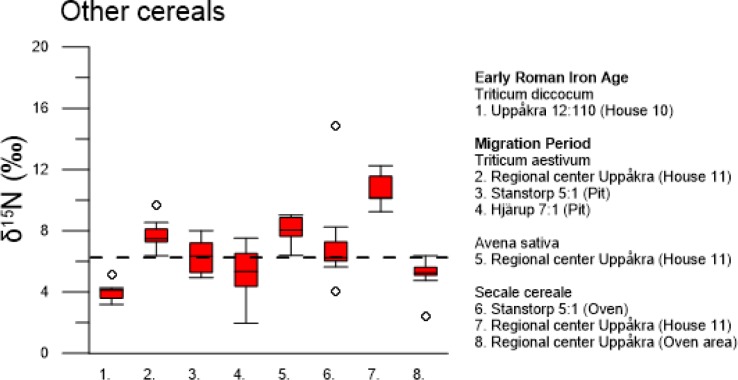
Stable nitrogen isotope values in grain of other cereal species from the archaeological study area. Emmer wheat, bread wheat, oat and rye from two archaeological periods: Early Roman Iron Age (AD 0–200) and Migration Period (AD 400–550). Dashed horizontal line at 6.3 ‰ indicates difference between low and high inputs of manure.

The δ^15^N values of charred grain from the archaeological study area are shown in Figs 4 and 5 and these represent periods in the first millennium AD. Summary results of isotopic analysis are presented in Tables 3 and 4 and individual results and context information are presented in S2 Table. For details of precision and accuracy, and calibration data, see S1 File.

**Table 3:** Summary of stable nitrogen isotope values in barley grain with statistical data from the archaeological study area. The Shapiro-Wilk value and Var.test refer to normal distribution and variability of archaeological samples compared to modern samples.

			δ^15^N (‰)					
Site	Sampled period	Sampled context	Mean	Variance	SD	Shapiro-Wilk (p-value)	Var.test (p-value)	Sample range (min-max)
**Main site**								
Regional center	ERIA	Hall-building stratum 12	9.85	17.33	4.16	0.9585	0.0001	15.2 (3.4–18.6)
	ERIA	House 110342 stratum 13a	4.3	4.98	2.23	0.9661	0.0789	7.8 (0.6–8.4)
	ERIA	Trench B	7.8	2.84	1.68	0.9400	0.3575	5.1 (4.8–9.9)
	LRIA	Hall-building stratum 7	5.38	1.15	1.07	0.9114	0.8762	4.2 (3.8–8.0)
	LRIA	House 110342 startum 9	7.54	2.42	1.56	0.9737	0.4698	5.3 (5.1–10.4)
	LRIA	House 56	6.78	0.84	0.92	0.9661	0.9485	3.3 (5.0–8.3)
	MP	Hall-building stratum 2	7.48	3.21	1.79	0.8440	0.2773	5.0 (4.4–9.4)
	MP	House 110342 stratum 2	10.99	12.22	3.5	0.9624	0.0008	12.9 (5.7–18.6)
	MP	Bårhuset	5.79	2.05	1.43	0.7531	0.5815	5.1 (4.5–9.6)
	MP	House 11	6.49	5.14	2.27	0.8432	0.0702	7.1 (4.4–11.5)
	MP	Pithouse 1	11.95	6.33	2.52	0.7996	0.0304	9.2 (5.3–14.5)
	MP	Hall-building House 24	7.07	4.19	2.05	0.7948	0.1388	5.9 (5.2–11.1)
	MP	House Vifot	6.28	0.32	0.57	0.9651	0.9982	1.9 (5.3–7.2)
	VP	Hall-building House 23	6.34	2.33	1.53	0.5805	0.4937	5.8 (5.0–10.8)
	VP	Oven area	9.89	6.75	2.6	0.9317	0.0228	8.0 (6.0–14.0)
	VIK	Hall-building House 22	7.86	6.58	2.57	0.8678	0.0256	9.2 (4.8–14.0)
	VIK	Ceremonial building	5.33	1.71	1.31	0.9648	0.6987	5.0 (2.9–7.9)
**Surrounding sites**								
Uppåkra 12:110	ERIA	House 5	7.52	5.43	2.33	0.8877	0.0573	6.7 (3.7–10.4)
Uppåkra 2:14	ERIA	Pithouse	3.36	0.91	0.95	0.7288	0.9350	3.8 (2.2–6.0)
Uppåkra 2:25	ERIA	House 17	5.05	0.71	0.84	0.9516	0.9698	2.7 (3.8–6.5)
Uppåkra 2:25	ERIA	House 31	6.05	1.83	1.35	0.9510	0.6558	4.6 (3.3–7.9)
Uppåkra 2:25	ERIA	Pit	3.28	2.66	1.63	0.8503	0.4023	4.9 (1.4–6.3)
Åttevägen	ERIA	Pit	7.31	4.87	2.21	0.9233	0.0852	6.4 (4.6–11.0)
Uppåkra 2:25	LRIA	House 4	8.49	0.54	0.73	0.9268	0.9880	2.2 (7.3–9.5)
Stanstorp 5:1	MP	Pit	7.58	2.07	1.44	0.9502	0.5776	5.0 (5.0–10.0)
Stanstorp 5:1	MP	Oven	7.83	3.33	1.83	0.8018	0.2549	4.5 (5.8–10.3)
Stanstorp 5:1	MP	Oven	8.63	10.38	3.22	0.7442	0.0023	10.6 (6.1–16.7)
Hjärup 7:1	MP	Pithouse	6.73	0.92	0.96	0.7975	0.9331	3.7 (5.6–9.3)
Hjärup 7:1	MP	Pit	7.48	0.76	0.87	0.9604	0.9628	2.8 (6.3–9.1)
Hjärup 21:36	VP	Pit	6.5	0.75	0.87	0.9276	0.9633	2.7 (4.8–7.5)
Hjärup 9:8	VIK	House 13	5.79	4.55	2.13	0.9203	0.1075	7.1 (3.0–10.1)
Hjärup 9:8	VIK	House 11	8.21	0.7	0.84	0.8436	0.9703	2.3 (7.0–9.3)

**Table 4:** Summary of stable nitrogen isotope values in grain of other cereal species with statistical data from the archaeological study area. The Shapiro-Wilk value and Var.test refer to normal distribution and variability of archaeological samples compared to modern samples.

				δ^15^N (‰)					
Site	Sampled period	Sampled context	Cereal species	Mean	Variance	SD	Shapiro-Wilk (p-value)	Var. test (p-value)	Sample range (min-max)
**Main site**	** **								
Regional center Uppåkra	MP	House 11	*T*. *aestivum*	7.69	0.76	0.87	0.5663	0.9629	3.2 (6.4–9.6)
	MP	House 11	*A*. *sativa*	8.00	0.69	0.83	0.4270	0.9720	2.6 (6.4–9.0)
	MP	House 11	*S*. *cereale*	10.54	0.89	0.94	0.3172	0.9385	3.1 (9.2–12.3)
	MP	Oven area	*S*. *cereale*	5.12	1.00	1.00	0.0095	^a^0.1908^a^	4.0 (2.4–6.4)
**Surrounding sites**									
Uppåkra 12:110	ERIA	House 10	*T*. *dicoccum*	4.04	0.27	0.52	0.7790	0.9991	2.0 (3.2–5.2)
Hjärup 7:1	MP	Pit	*T*. *aestivum*	5.53	4.45	2.11	0.6412	0.1154	6.7 (2.0–8.7)
Stanstorp 5:1	MP	Pit	*T*. *aestivum*	6.05	1.31	1.14	0.6267	0.8310	3.6 (4.4–8.0)
	MP	Oven	*S*. *cereale*	7.10	7.76	2.79	0.0014	^a^0.5229^a^	10.8 (4.1–14.9)

^a^ A modified Levene's test was utilised as the Shapiro-Wilk’s test rejected the null-hypothesis of normality.

#### Manure intensity

The modified Wilcox test shows a significant effect of time period (p < 0.0001), but no significant difference between regional center Uppåkra and the surrounding sites. The pairwise t-tests show that the mean δ^15^N values from the Early Roman Iron Age (AD 0–200) differed significantly from the other periods (AD 200–1000). There were, however, no significant differences between the other periods. This indicates that the manuring intensity on average was lower in the study area during the Early Roman Iron Age compared to the following periods.

**Fig 6:**
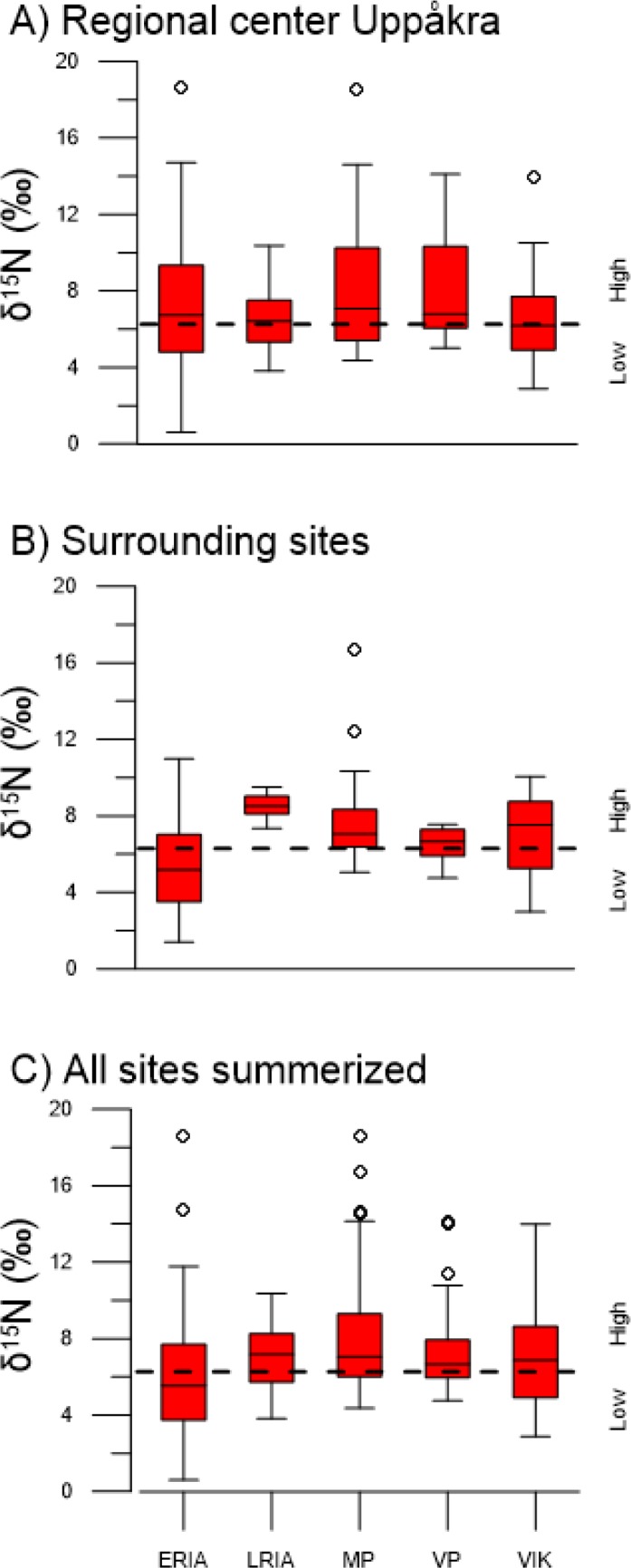
Stable isotope values in barley grain summarized for individual archaeological periods. Summarized results from a) regional center Uppåkra, b) surrounding sites and c) all sites combined from the study area. Dashed horizontal line at 6.3 ‰ indicates difference between low and high inputs of manure.

The modified Wilcox test of δ^15^N in barley grain from the archaeological sites, together with the distinction of low and high inputs of manure at 6.3 ‰ from the field experiment, provide indications for past manure practices. Pooled isotopic values from barley grain per period are presented in Fig 6 for the regional center Uppåkra, surrounding sites and from the study area combined, respectively. The pooled data in the study area indicate that the manure rates were low to medium during the Early Roman Iron Age and medium to high in the other periods. Some individual samples in the periods after AD 200 show exception to the overall higher values, with lower values in contexts 4, 9, 14 and 17 at the regional center (see Fig 4). The observed variation between different sample ranges are within the same overall isotopic range, and there is no significant difference in manuring intensity between regional center Uppåkra and the surrounding sites.

δ^15^N values of grain from other cereal species were determined in two periods: Early Roman Iron Age and Migration Period (Fig 5). We have chosen to compare the measured δ ^15^N values in ancient grain from bread wheat, emmer wheat, rye and oat with values obtained from modern hulled barley in the field experiment, as we have no references of δ ^15^N values in grain for the other cereals. The use of the modern barley δ ^15^N values to interpret the other cereal grain δ ^15^N values was motivated by the fact that other modern studies found no difference in the effect of manuring on different cereal species, e.g. Fraser et al. [30]. The low δ^15^N value in grain of emmer wheat during the Early Roman Iron Age is similar to those in the lower range of hulled barley. In the Migration Period, both bread wheat and rye show varied δ^15^N values in grain, ranging from low to high. A sample of oat had high δ^15^N values.

#### Single and multiple locations

The variance observed in the field experiment within the plot receiving manure (1. A0) is used to explore if grains from an archaeological context originated from a single location or multiple locations. The results of the F-tests are presented in Table 3 together with the p-values of the Shapiro-Wilk’s test. As this is an exploratory study with rather small sample numbers, we have decided to use a significance level of 10% when interpreting the results. The Shapiro-Wilk’s test turns out to be non-significant for the δ^15^N measurement in all the samples, which means that the δ^15^N values are normally distributed. From the regional center site, the contexts Hall-building (stratum 12), House 110342 (stratum 13a), House 110342 (stratum 2), House 11, Pithouse 1, Hall-building (House 22) and Oven area, have significantly greater variance than the reference plot, consistent with grains derived from multiple locations. The archaeological interpretations of these depositional contexts are expected to have been formed during a single event and thus likely representing grain accumulated from a single year.

From the surrounding sites only contexts at House 5 (Uppåkra 12:110), Pit A459 (Åttevägen), and Oven PM48950 (Stanstorp 5:1) have significantly greater variances than the reference, consistent with grains derived from multiple locations. The oven context in House 13 (Hjärup 9:8) is borderline with a p-value of 0.1075. Here the archaeological interpretations are also thought to represent contexts with grain accumulated during a single year. Some interpretive caution should, however, be taken for grains around the oven features, as grain may have been accumulated over multiple years if the oven was in use for a longer time.

The results of the F-tests and the p-values of the Shapiro-Wilk’s test for the other cereals species are presented in Table 4. As we had no reference values for variance in δ^15^N for these cereals, we chose to compare the observed variances with the variances from the hulled barley field experiment again using the plot receiving manure (1. A0). None of the samples had δ^15^N variances significantly greater than the hulled barley field experiment sample and this is consistent with grains of non-barley cereal species derived from single locations.

**Fig 7:**
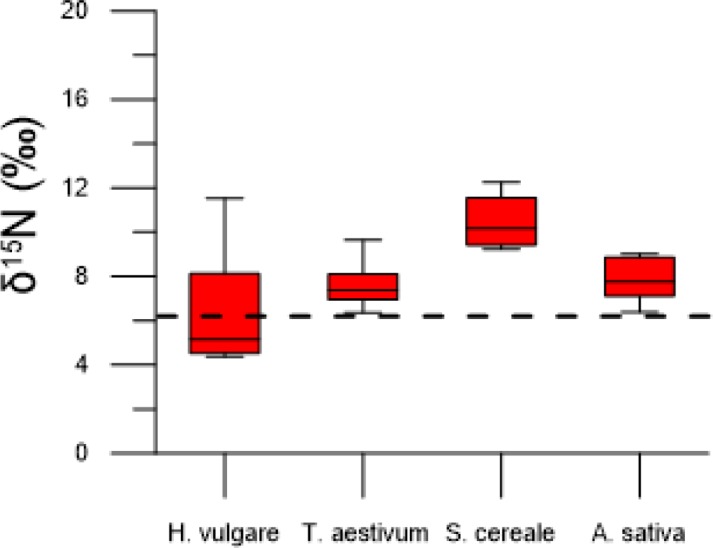
Stable nitrogen isotope values in grain from House 11 at regional center Uppåkra. Dashed horizontal line at 6.3 ‰ indicates difference between low and high inputs of manure.

Additional insight into manuring practices between cereal species was possible in grain from the same context in House 11 from regional center Uppåkra. A charcoal/ash-rich layer on top of a clay floor, formed after a house-fire, contained grain from four different cereal species: hulled barley, bread wheat, rye and oat. The deposited grain in the layer are likely cereals handled in the household during the same year. Hulled barley showed the widest isotopic range, with low to high values, whereas the other three cereal species had narrow isotopic ranges with high values (Fig 7). Furthermore, the observed variance (see above) of the hulled barley sample is consistent with grain derived from multiple locations, whereas the other three cereal species are consistent with grain derived from single locations.

## Discussion

### Manuring intensity in crop production

The data from the modern field experiment show a clear difference in δ^15^N values of grain from non-manured growing conditions compared to manured treatment. Long-term absence of nutritional input showed lower δ^15^N values in barley grain compared to high δ^15^N values in grain with manured treatment. Other long-term experiments of cereal cultivation have similarly shown that after prolonged absence of nutritional input to the arable land, δ^15^N values of grain significantly drop [30,62].

The existence of the regional center Uppåkra throughout the first millennium AD, a populous settlement with smaller groups of farms in its surrounding areas, would have put great stress on arable land over time due to its demand for grain supplies. No difference between Uppåkra and surrounding sites was observed in δ^15^N values of grain to suggest that agricultural practices using manure differed, with the exception for rye and bread wheat. These latter cereals have higher δ^15^N values at the regional center compared to the surrounding sites, but should be interpreted with caution, as the data derive from few samples. At present, these are the only Iron Age sites in Sweden from which charred grain have been isotopically analyzed; it is therefore not possible to compare the manuring intensity of the study area to other Iron Age sites in nearby regions.

The analysis of nitrogen isotopes of barley grain show a large range in δ^15^N values during the early phase of the sites in the study area (AD 0–200). It indicates variation in manuring levels between cultivated fields. This could reflect how manure was a limited resource at the time when the regional center Uppåkra and several smaller settlements were established and application of manure varied between arable lands as new areas were opened up for crop cultivation. The overall high δ^15^N values of barley grain in the following periods (AD 200–1000) suggests more intensive manuring.

The lower δ^15^N values observed from a few contexts at the regional center Uppåkra during the periods after AD 200 could indicate an expansion in arable cultivation to marginal areas. Crops cultivated in fields further from the settlements and penning areas may have had less additional manuring inputs and instead relied on fallow land with only grazing animals, contributing to low-level manuring, to replenish soil nutrients. Transport of manure would have involved high labor inputs and manure is generally spread within arable land adjacent to settlements or stalling areas [63]. For that reason, peripheral fields may not have received the same routine addition of manure and this may explain the lower δ^15^N values in grain.

The δ^15^N values in grain from the other cereal species (bread wheat, emmer wheat, rye and oat) are within the same range as barley δ^15^N values, with no clear distinction in cultivation practices between different crops. Only in House 11 at the regional center, rye has higher δ^15^N values than the other cereal species. This suggests that applications of manure in crop cultivation was overall similar among cereal species in the study area as no species could be identified to have a typical low or high isotopic range, except rye in House 11. Alternatively, the minor crops could have been cultivated in the same fields as were used for barley, thus showing similar manuring levels to barley. Several other studies on prehistoric crop cultivation have, on the contrary, shown that the manuring rate differed between cereal species and explained the difference between individual species as reflecting their economic importance in the society [17,27]. A study by Styring et al. [27] from Iron Age south-west Germany revealed that barley was manured more than other cereal species. It was suggested that intensive manuring of barley, used in beer production, could be linked to the political importance of drinking and feasting in the Iron Age society and that this was reflected in crop husbandry practices. Signs of the economic status of different crops can additionally be supported from the archaeobotanical record of the study area, which shows hulled barley to be the dominant cereal throughout the first millennium AD [64]. That barley was the principal cereal in southern Scandinavia is evidenced by its common representation on other sites in southern Sweden [12,65,66], but it appears particularly dominant in the study area. The economic importance of barley could therefore reflect, similar to the study by Styring et al. [27], its value in the Scandinavian Iron Age society with a cultural importance of beer. Moreover, evidence of malt from barley grain in the processes to make beer has been recovered around kilns at the regional center Uppåkra, likely aimed for large-scale production [45]. It is therefore interesting that there is no isotopic evidence for more intensive manuring of barley in this study region.

Although isotopic results published by Gron et al. [17] have revealed that manuring was already practiced in southern Sweden in the Early Neolithic, farming was likely performed on a small-scale and inputs of manure were suggested to have been in limited supply, as is often supposed for that period in Northern Europe [9]. If the common representation of barley in the study area reflects surplus production, increased scale of cultivation could have involved more intensive manuring. Furthermore, with no decrease in grain δ^15^N values that indicates a reduction in the intensity of manuring during AD 200–1000, we suggest that manuring levels were maintained, reflecting ample availability of manure.

Several δ^15^N values of grain, particularly in the Early Roman Iron Age and in the Migration Period, are either significantly below or significantly above the range of either of the ranges for the experimental study’s manured and non-manured plots. Within agricultural systems using manure, the nitrogen isotope composition of the crop being cultivated is influenced by the nitrogen isotope composition of the fertilizer applied [67]. The use of fertilizers will affect plant δ^15^N values by a variable amount depending on fertilizer used (e.g., animal manure, compost, by-products of animal origin, wood ash etc.), amount applied, frequency and duration of application [19]. Fertilizers´ influence on the N isotopic composition of plants will also be dependent on how the particular fertilizer affects transformations and losses of N, since these processes can discriminate against ^15^N [68]. With respect to animal manure, pig and cattle are the two most common species in the zooarchaeological record in the study area [69]. It is therefore likely that dung from these animal species was an integral part of the manures applied to the arable fields. The δ^15^N values of pig manure is typically higher than δ^15^N values of cattle manure [67]. If the routine of applying manure from these animals differed, e.g. more intensive application of pig manure, this may have influenced some archaeological grain assemblages to have higher δ^15^N values than grain from the agricultural experiment using manure from cattle. Moreover, the δ^15^N values of animal manure tend to be affected by various stages prior to application on the arable fields (collection, composting, and storage) and decomposition after application, due to nitrogen losses particularly from NH_3_ volatilization and denitrification [19,29,70]. Accordingly, manure δ^15^N may increase during these stages. Manuring practices may have, on one hand, varied between sites and over time in the study area, but if practices were different to the conditions in the field experiment, this may account for some δ^15^N values of the ancient grain to be anomalously low and high compared to grain from the field experiment.

### Crops from single and multiple harvesting sites

The wide ranges and large variance observed in grain δ^15^N values from contexts at the regional center Uppåkra and on four surrounding sites indicate that the crops may not have been gathered from a single harvest site. Intra-sample variability of δ^15^N in archaeological cereal grains can be high due to some variation in growing conditions, possibly through crops harvested from the same location over multiple years or crops gathered from multiple locations in the same year [13].

The archaeological contexts of barley samples with large δ^15^N ranges and variance at the regional center Uppåkra are from floor deposits formed after house-fires, except at the oven area. The depositional contexts are interpreted to represent *in situ* remains of the domestic space, including grain from a single year [6]. The samples are from grain-rich assemblages, likely of stored grain, contrary to the otherwise common finds of smaller quantities of grain, accumulated as spill when handled in houses. It is unclear if the large variance observed in samples from oven structures and postholes from the surrounding sites accumulated during a single or multiple years. A refuse pit with a layer containing grain has been interpreted to be waste from one seasonal use, and is the only crop assemblage among the surrounding sites that is consistent with grains gathered from multiple locations in a single year. Large δ^15^N ranges and variance were not observed in the other cereal species (bread wheat, emmer wheat, rye and oat).

Samples with large δ^15^N ranges and variance, given their archaeological context, are likely of crops gathered over a single year from multiple locations. Whether this reflects how the regional center Uppåkra managed multiple fields with different manuring rates for its cultivation of barley or collected grain from the surrounding farming community to meet its demand for grain, or both, is not possible to say. Nor is it possible to say if the large variance observed from samples from the surrounding settlements is from exchange with crops between farmers or if multiple fields were used by farmsteads for barley cultivation. It indicates, however, that grain assemblages gathered from different harvesting sites were pooled together and stored, which suggests that cultivation of barley involved multiple fields intended for large-scale production. Moreover, the isotopic data from crop assemblages of barley, bread wheat, rye and oat in House 11 at regional center Uppåkra showed that only barley had large δ^15^N range and variance, providing additional data that is consistent with barley grain gathered from different harvesting sites. This further emphasizes the economic importance of barley, and possibly mobility of surplus production of barley within the study area. Samples with small δ^15^N ranges and variance are on the contrary, consistent with crops gathered from single harvesting sites, suggesting household level of production and storage.

Several studies on zooarchaeological data from the same study area have discussed transportation of animals and meat supplies. In a study by Price [71] on animal teeth from regional center Uppåkra, strontium isotope analysis showed largely local provenience for pig and cattle bone, but some cattle showed non-local values. The occurrence of non-local values of cattle was explained by import of animals to the site. Similar to the non-local strontium isotopic signatures of livestock, other zooarchaeological studies noted indications for meat produce having been transported within the study area. Studies from three of the surrounding sites–Uppåkra 12:110, Uppåkra 2:25 and Hjärup 21:36 –have found that meat-rich parts of animals were significantly underrepresented among the bone assemblages, regardless of species [49,51,72]. It was suggested that meat-rich parts were “exported” elsewhere, likely as food supplies to regional center Uppåkra. The osteological research, paired with the isotopic analysis of crop remains, points to mobility of agricultural food products used by the communities in the study area.

### Agricultural wealth and crop production

In the Scandinavian Iron Age society, the regional center Uppåkra show imprints of extraordinary character by its scale of material affluence, skilled artisan production and implications for wide networks of trade [73–80]. The site’s long continuity together with the size of the settlement and the large-scale consumption sets it apart from most other contemporary settlements and raises questions about its food supplies. The regional center may have had a great demand for staple grain and depended on intensive cultivation. The area of land that must have been under cultivation and manured was perhaps greater, and therefore required more investment. In such a populous community, fertilizers could have become a limiting factor in the use of agricultural land. The isotopic data from the regional center and its surrounding areas offers an approach to investigate manuring practices the growers used in the production of crops for optimizing the yield.

Diversification and increased production (surplus) by farmers could be a means to ensure sufficient food supplies [81]. Crop diversity would ensure production yield should a particular crop fail. The wide range of cereal species present in the archaeobotanical record from the study area indicates diverse production of cereals [42,64,82]. Surplus production is more difficult to examine from archaeological records as intensive cultivation can involve both increased labor and resource input, and expansion of cultivated land [27,83]. The trend of high δ^15^N values in barley grain from the study area during AD 200–1000 and barley as the principal crop across the first millennium AD point to surplus crop production achieved by intensive and sustained use of manure. Manure input similar to barley was found also among other cereal species, and it suggests systematic use of manure in cereal production. This was not found to be the case in manuring practices among farmers in the early Neolithic in southern Sweden. Gron et al. [17] suggested that manure was in limited supply in cereal cultivation and that manure was selectively applied between cereal species.

Long-term use of permanent fields involving intensive management by the routine of adding nutrition to the soil implies conscious and planned manipulation of arable lands. The consistent finds of seeds from typical arable weeds, such as fat hen (*Chenopodium album*), black-bindweed (*Fallopia convolvulus*) and redshank (*Persicaria maculosa*), in archaeobotanical samples from the study area forms another set of data indicating cultivation on manured soils [42,64]. Such weeds are typical in crop assemblages sampled for isotopic analysis in the present study, but they are also widely common among crop assemblages in the study area across time and support also long-term use of permanent fields.

Cultivators likely used variable organic matter as manures, including the use of both domestic waste and animal dung to maintain and enhance crop yield. Spreading domestic waste from small-scale households on arable land was likely insufficient as intensive fertilizer across areas required to produce staple grain crops [9]. Animal manure may therefore have played a potential role in crop production. Cattle could possibly have been of particular importance as they are a good provider of manure in bulk among farm animals.

Use of animal manure in crop cultivation also has wider implications for animal husbandry. The isotopic data in this study indicate that sustained and high manuring levels were maintained in cereal cultivation and that inputs were applied across species. This points to ample availability of manure and could reflect an agricultural aspect of the wealth of Uppåkra and its surrounding areas, since intensive manuring would have required livestock ownership and high labor inputs. This is consistent with the zooarchaeological data across the study area where rich quantities of bone from livestock has been found, predominantly those of cattle, followed by pig [69,72,84]. Structures from stalls or enclosures to estimate the scale of livestock at individual sites have not been found from the study area. Data on charcoal, combined with ecological signals from seeds, can alternatively contribute to insights on land management linked to animal husbandry. Charcoal analysis from the regional center Uppåkra [85] has shown indications for high proportions of branch wood (ash, hazel and oak) and a low frequency of trunk wood, suggesting the use of leaf fodder, and has been interpreted to be linked to the keeping of animals. Widespread distribution of seeds from plants typical for pasture or meadow vegetation in house structures on sites in the study area has been found from previous archaeobotanical analysis, indicating handling of fodder and that animal husbandry played an important role in the agrarian economy [42,64]. This is consistent also with the palynological record of southern Sweden, which shows that the landscape in the Iron Age was a period with continued expansion of pastures and crop cultivation, initiated during the Late Bronze Age [86].

There was overall no difference in cereal grain δ^15^N values between regional center Uppåkra and the surrounding sites. This suggest that manuring practices in crop cultivation were much the same among settlements in the study area. Alternatively, exchange of staple grain could have existed between settlements causing mobility of staple grain in the area, with the possibility that surrounding settlements were involved in supplying the regional center with grain. A system with crop production moving from the periphery to Uppåkra was suggested in another archaeobotanical study from the same sites by the observed size difference in barley grain [44]. Large, high-quality grain was more frequent at the regional center Uppåkra, primarily at a few house contexts in a central area of the site, when compared to sites in the surrounding areas, where smaller grain size was more common. This observed difference in grain size was suggested to reflect selection for high-quality grain after crop processing was completed, possibly intended for beer production or grain for seeding. Given the powerful status and administrative role the regional center is believed to have had in the region [3], it was suggested that the regional center could have had a role in accessing agrarian products among farmers by collecting, storing and redistributing grain in the agricultural community. Although crop production can be regarded as predominantly aimed at satisfying the needs of peasant households themselves, it is conceivable that economic relations concerning cereal produce existed between the regional center and nearby settlements. Tentatively, this could explain why the cereal grain δ^15^N values are similar from the regional center and the surrounding sites. The intra-sample variability of barley grain δ^15^N values from several contexts in the study area, consistent with stored barley from multiple harvesting sites could similarly point to economic relations in crop production between different producers or settlements.

We envisage that the trend of overall high δ^15^N values of grain across time in the study area reflect sustained resource input to maintain intensive cereal cultivation needed to meet the demand for stable grain at the regional center Uppåkra. With respect to intensive manuring and more investments in crop production, it diversifies the affluence ascribed to the regional center Uppåkra to include an aspect of prosperity also in the agricultural production at the base of the society.

## Conclusions

The results from the nitrogen isotope analysis of modern barley in the field experiment show a clear difference in grain δ^15^N values from growing conditions with long-term absence of manuring compared to manured treatment. It has provided an interpretive framework that allows us to compare values from known growing conditions with archaeological grain from sites in the study area to explore past manuring practices and intensity in arable production.

The isotopic results from contexts at the regional center Uppåkra and from a set of surrounding sites show that manuring inputs in crop cultivation varied during the initial period (AD 0–200), but were overall high during the later periods (AD 200–1000). No difference in manure input was found between barley grains handled at the regional center Uppåkra compared to those on the surrounding sites. The isotopic data from bread wheat, emmer wheat, rye and oat show that these minor crops were cultivated on arable land with manure input similar to barley, indicating manure was a resource in ample supply for cultivation across cereal species. The results show further that bread wheat and rye at the regional center had higher δ^15^N values compared to the surrounding sites. We suggest the long-term trend for high manuring intensity indicates systematic and sustained use of manure.

The nitrogen isotopic composition of barley from several cereal assemblages is consistent with grains grown in different conditions, indicating that barley grain may have been harvested from multiple locations and then pooled and stored together. This builds on existing knowledge of barley production by demonstrating that grain production of barley was not only large-scale, but that consumers could have relied on multiple areas of arable land for its production. Moreover, the long-term trend of crop cultivation on manured soils, evidenced from high δ^15^N values in grain, signals agricultural wealth in the study area and this pairs with the economic affluence that is ascribed to the regional center Uppåkra. The sustained and productive crop growing conditions probably also contributed to the unusually long settlement continuity. We find that the prosperity of the plant economy diversifies our understanding of the affluence and settlement continuity at the regional center Uppåkra and the isotopic results link wealth to its agricultural base.

## Supporting information

S1 File Supplementary results. Details of precision and accuracy, and calibration data (δ^15^N and %N).
(DOCX)

S1 Table Dataset of modern grain from field experiment. δ^15^N values, sample information and metric data for all analyzed grain.
(XLSX)

S2 Table Dataset of archaeological grain from study area. δ^15^N values, sample information, metric data and archaeological accession information for all analyzed grain.
(XLSX)
