# Supplementary material for: Manuring practices in the first millennium AD in southern Sweden inferred from isotopic analysis of crop remains
Source: PLoS One. 2019 Apr 18;14(4):e0215578. doi: 10.1371/journal.pone.0215578 (PMC6472791; doi:10.1371/journal.pone.0215578)
Supplement: S1 File — Details of precision and accuracy, and calibration data (δ15N and %N). (DOCX) [file pone.0215578.s001.docx]

Precision of SRM’s for the analysis of ancient grains:

The standard deviation for δ^15^N calibration on the basis of 41 measurements of USGS40 (given value of δ^15^N -4.5 ‰ AIR) was 0.1 ‰ and 31 measurements of USGS41a (given value of δ^15^N 47.5 ‰ AIR) was 0.1 ‰.

The standard deviation for %N calibration on the basis of 41 measurements of USGS40 (given value of 9.52 % N) was 0.38 % and 31 measurements of USGS41a (given value of 9.52% N) was 0.44 %.

Precision of QC Check Samples for the analysis of ancient grains:

The standard deviation for δ^15^N on the basis of 21 measurements of GA1 QC (given value of δ^15^N -5.2 ‰ AIR) was 0.1 ‰ and 21 measurements of GA2 QC (given value of δ^15^N -3.1 ‰ AIR) was 0.1 ‰.

The standard deviation for %N on the basis of 21 measurements of GA1 QC (given value of 9.52 % N) was 0.43 % and 21 measurements of GA2 QC (given value of 9.52 % N) was 0.25 %.

Precision of duplicate analyses of ancient grains:

The average standard deviation for δ^15^N on the basis of 42 duplicate measurements of 400 ancient grains samples was 0.1 ‰.

The average standard deviation for %N on the basis of 42 duplicate measurements of 400 ancient grains samples was 0.02 %.

Accuracy of QC Check Samples for the analysis of ancient grains:

The average result for δ^15^N on the basis of 21 measurements of GA1 QC was δ^15^N -5.2 ‰ AIR (given value of δ^15^N -5.2 ‰ AIR) and 21 measurements of GA2 QC was δ^15^N -3.1 ‰ AIR (given value of δ^15^N -3.1 ‰ AIR).

The average result for %N on the basis of 21 measurements of GA1 QC was 9.48 % (given value of 9.52 % N) and 21 measurements of GA2 QC was 9.64 % (given value of 9.52 % N).

Precision of SRM’s for the analysis of modern grains:

The standard deviation for δ^15^N calibration on the basis of 16 measurements of USGS40 (given value of δ^15^N -4.5 ‰ AIR) was 0.2 ‰ and 12 measurements of USGS41a (given value of δ^15^N 47.6 ‰ AIR) was 0.1 ‰.

The standard deviation for %N calibration on the basis of 16 measurements of USGS40 (given value of 9.52 % N) was 0.60 % and 12 measurements of USGS41a (given value of 9.52 % N) was 0.15 %.

Precision of QC Check Samples for the analysis of modern grains:

The standard deviation for δ^15^N on the basis of 8 measurements of GA1 QC (given value of δ^15^N -5.2 ‰ AIR) was 0.1 ‰ and 8 measurements of GA2 QC (given value of - δ^15^N 3.1 ‰ AIR) was 0.1 ‰.

The standard deviation for %N on the basis of 8 measurements of GA1 QC (given value of 9.52 % N) was 0.43 % and 8 measurements of GA2 QC (given value of 9.52 % N) was 0.09 %.

Precision of duplicate analyses of modern grains:

The average standard deviation for δ^15^N on the basis of 8 duplicate measurements was 0.1 ‰.

The average standard deviation for %N on the basis of 18 duplicate measurements was 0.02 %.

Accuracy of QC Check Samples for the analysis of modern grains:

The average result for δ^15^N on the basis of 8 measurements of GA1 QC was δ^15^N -5.2 ‰ AIR (given value of δ^15^N -5.2 ‰ AIR) and 8 measurements of GA2 QC was δ^15^N -3.1 ‰ AIR (given value of δ^15^N -3.1 ‰ AIR).

The average result for %N on the basis of 8 measurements of GA1 QC was 9.48 % (given value of 9.52 % N) and 8 measurements of GA2 QC was 9.58 % (given value of 9.52 % N).

| **Calibration Data - Modern Grain 15N Analysis** | | | | | |  |  |
| --- | --- | --- | --- | --- | --- | --- | --- |
|  |  |  |  |  |  |  |  |
| **SRM/QC** | **15N** | **%N** | **RunNo** |  |  |  |  |
|  |  |  |  |  |  |  |  |
|  |  |  |  |  | **USGS40** | **15N** | **%N** |
| 40 | -4,43 | 9,18 | 171027DA |  | **given** | -4,52 | 9,52 |
| 40 | -4,24 | 9,27 | 171027DA |  | **average** | -4,52 | 9,38 |
| 40 | -4,60 | 9,54 | 171027DA |  | **stdev** | 0,18 | 0,60 |
| 40 | -4,81 | 9,49 | 171027DA |  | **upper dev from average** | 0,28 | 0,56 |
| 40 | -4,38 | 9,58 | 171027DB |  | **lower dev from average** | -0,29 | -2,13 |
| 40 | -4,52 | 9,56 | 171027DB |  | **confidence interval 95%** | 0,09 | 0,30 |
| 40 | -4,42 | 7,24 | 171027DB |  | **count** | 16 | 16 |
| 40 | -4,75 | 9,54 | 171027DB |  |  |  |  |
| 40 | -4,34 | 9,22 | 171027DC |  | **USGS41a** | **15N** | **%N** |
| 40 | -4,37 | 9,70 | 171027DC |  | **given** | 47,55 | 9,52 |
| 40 | -4,74 | 9,50 | 171027DC |  | **average** | 47,57 | 9,61 |
| 40 | -4,63 | 9,50 | 171027DC |  | **stdev** | 0,13 | 0,15 |
| 40 | -4,29 | 9,94 | 171027DD |  | **upper dev from average** | 0,26 | 0,30 |
| 40 | -4,44 | 9,85 | 171027DD |  | **lower dev from average** | -0,21 | -0,22 |
| 40 | -4,57 | 9,57 | 171027DD |  | **confidence interval 95%** | 0,07 | 0,08 |
| 40 | -4,78 | 9,37 | 171027DD |  | **count** | 12 | 12 |
|  |  |  |  |  |  |  |  |
| 41a | 47,52 | 9,40 | 171027DA |  | **GA1 QC** | **15N** | **%N** |
| 41a | 47,61 | 9,59 | 171027DA |  | **given** | -5,22 | 9,52 |
| 41a | 47,58 | 9,53 | 171027DA |  | **average** | -5,22 | 9,37 |
| 41a | 47,50 | 9,58 | 171027DB |  | **stdev** | 0,11 | 0,44 |
| 41a | 47,83 | 9,67 | 171027DB |  | **upper dev from average** | 0,19 | 0,31 |
| 41a | 47,38 | 9,72 | 171027DB |  | **lower dev from average** | -0,19 | -1,07 |
| 41a | 47,49 | 9,77 | 171027DC |  | **confidence interval 95%** | 0,08 | 0,31 |
| 41a | 47,63 | 9,65 | 171027DC |  | **count** | 8 | 8 |
| 41a | 47,58 | 9,53 | 171027DC |  |  |  |  |
| 41a | 47,71 | 9,91 | 171027DD |  | **GA1 QC** | **15N** | **%N** |
| 41a | 47,64 | 9,59 | 171027DD |  | **given** | -3,07 | 9,52 |
| 41a | 47,36 | 9,39 | 171027DD |  | **average** | -3,08 | 9,58 |
|  |  |  |  |  | **stdev** | 0,11 | 0,09 |
| GA1 QC | -5,03 | 9,31 | 171027DA |  | **upper dev from average** | 0,22 | 0,14 |
| GA1 QC | -5,28 | 9,53 | 171027DA |  | **lower dev from average** | -0,12 | -0,13 |
| GA1 QC | -5,21 | 9,49 | 171027DB |  | **confidence interval 95%** | 0,07 | 0,07 |
| GA1 QC | -5,14 | 9,55 | 171027DB |  | **count** | 8 | 8 |
| GA1 QC | -5,24 | 9,55 | 171027DC |  |  |  |  |
| GA1 QC | -5,42 | 9,54 | 171027DC |  |  |  |  |
| GA1 QC | -5,24 | 9,68 | 171027DD |  |  |  |  |
| GA1 QC | -5,22 | 8,30 | 171027DD |  |  |  |  |
|  |  |  |  |  |  |  |  |
| GA2 QC | -2,86 | 9,45 | 171027DA |  |  |  |  |
| GA2 QC | -3,10 | 9,53 | 171027DA |  |  |  |  |
| GA2 QC | -3,10 | 9,59 | 171027DB |  |  |  |  |
| GA2 QC | -3,04 | 9,61 | 171027DB |  |  |  |  |
| GA2 QC | -3,20 | 9,61 | 171027DC |  |  |  |  |
| GA2 QC | -3,21 | 9,68 | 171027DC |  |  |  |  |
| GA2 QC | -3,12 | 9,72 | 171027DD |  |  |  |  |
| GA2 QC | -3,05 | 9,46 | 171027DD |  |  |  |  |

| **Calibration Data - Ancient Grain 15N Analysis** | | | | | |  |  |
| --- | --- | --- | --- | --- | --- | --- | --- |
|  |  |  |  |  |  |  |  |
| **SRM/QC** | **15N** | **%N** | **RunNo** |  |  |  |  |
|  |  |  |  |  |  |  |  |
| 40 | -4,41 | 9,46 | 170503DA |  | **USGS40** | **15N** | **%N** |
| 40 | -4,54 | 9,39 | 170503DA |  | **given** | -4,52 | 9,52 |
| 40 | -4,58 | 9,47 | 170503DA |  | **average** | -4,52 | 9,57 |
| 40 | -4,54 | 9,27 | 170503DA |  | **stdev** | 0,12 | 0,29 |
| 40 | -4,32 | 9,58 | 170503DB |  | **upper dev from average** | 0,27 | 1,45 |
| 40 | -4,42 | 9,56 | 170503DB |  | **lower dev from average** | -0,25 | -0,81 |
| 40 | -4,69 | 9,59 | 170503DB |  | **confidence interval 95%** | 0,02 | 0,06 |
| 40 | -4,66 | 9,46 | 170503DB |  | **count** | 101 | 101 |
| 40 | -4,28 | 9,72 | 170503DC |  |  |  |  |
| 40 | -4,60 | 9,50 | 170503DC |  | **USGS41a** | **15N** | **%N** |
| 40 | -4,70 | 9,48 | 170503DC |  | **given** | 47,55 | 9,52 |
| 40 | -4,49 | 9,46 | 170503DC |  | **average** | 47,57 | 9,65 |
| 40 | -4,33 | 9,63 | 170503DD |  | **stdev** | 0,13 | 0,34 |
| 40 | -4,62 | 9,70 | 170503DD |  | **upper dev from average** | 0,29 | 1,72 |
| 40 | -4,62 | 9,81 | 170503DD |  | **lower dev from average** | -0,41 | -0,73 |
| 40 | -4,50 | 9,34 | 170503DD |  | **confidence interval 95%** | 0,03 | 0,08 |
| 40 | -4,47 | 9,64 | 170503DE |  | **count** | 76 | 76 |
| 40 | -4,50 | 9,48 | 170503DE |  |  |  |  |
| 40 | -4,52 | 9,55 | 170503DE |  | **GA1 QC** | **15N** | **%N** |
| 40 | -4,60 | 9,47 | 170503DE |  | **given** | -5,22 | 9,52 |
| 40 | -4,45 | 9,43 | 170615DA |  | **average** | -5,22 | 9,48 |
| 40 | -4,50 | 9,50 | 170615DA |  | **stdev** | 0,09 | 0,42 |
| 40 | -4,61 | 9,51 | 170615DA |  | **upper dev from average** | 0,21 | 0,85 |
| 40 | -4,51 | 9,54 | 170615DA |  | **lower dev from average** | -0,20 | -1,51 |
| 40 | -4,39 | 9,59 | 170615DB |  | **confidence interval 95%** | 0,03 | 0,12 |
| 40 | -4,55 | 9,59 | 170615DB |  | **count** | 51 | 51 |
| 40 | -4,70 | 9,47 | 170615DB |  |  |  |  |
| 40 | -4,44 | 9,50 | 170615DB |  | **GA1 QC** | **15N** | **%N** |
| 40 | -4,25 | 9,65 | 170615DC |  | **given** | -3,07 | 9,52 |
| 40 | -4,55 | 9,69 | 170615DC |  | **average** | -3,07 | 9,62 |
| 40 | -4,68 | 9,94 | 170615DC |  | **stdev** | 0,09 | 0,20 |
| 40 | -4,59 | 10,77 | 170615DC |  | **upper dev from average** | 0,15 | 0,74 |
| 40 | -4,44 | 9,43 | 170615DD |  | **lower dev from average** | -0,19 | -0,25 |
| 40 | -4,50 | 9,59 | 170615DD |  | **confidence interval 95%** | 0,03 | 0,06 |
| 40 | -4,51 | 9,55 | 170615DD |  | **count** | 51 | 51 |
| 40 | -4,63 | 9,49 | 170615DD |  |  |  |  |
| 40 | -4,47 | 9,58 | 170615DE |  |  |  |  |
| 40 | -4,48 | 9,65 | 170615DE |  |  |  |  |
| 40 | -4,45 | 9,49 | 170615DE |  |  |  |  |
| 40 | -4,68 | 9,39 | 170615DE |  |  |  |  |
| 40 | -4,50 | 9,49 | 170615DF |  |  |  |  |
| 40 | -4,36 | 9,48 | 170615DF |  |  |  |  |
| 40 | -4,64 | 9,48 | 170615DF |  |  |  |  |
| 40 | -4,59 | 9,47 | 170615DF |  |  |  |  |
| 40 | -4,48 | 9,20 | 170615DG |  |  |  |  |
| 40 | -4,46 | 9,30 | 170615DG |  |  |  |  |
| 40 | -4,58 | 9,57 | 170615DG |  |  |  |  |
| 40 | -4,56 | 9,61 | 170615DG |  |  |  |  |
| 40 | -4,51 | 9,51 | 170615DH |  |  |  |  |
| 40 | -4,50 | 9,51 | 170615DH |  |  |  |  |
| 40 | -4,48 | 9,55 | 170615DH |  |  |  |  |
| 40 | -4,59 | 9,49 | 170615DH |  |  |  |  |
| 40 | -4,44 | 9,64 | 170615DI |  |  |  |  |
| 40 | -4,36 | 9,64 | 170615DI |  |  |  |  |
| 40 | -4,71 | 9,91 | 170615DI |  |  |  |  |
| 40 | -4,56 | 9,34 | 170615DI |  |  |  |  |
| 40 | -4,47 | 9,49 | 170615DJ |  |  |  |  |
| 40 | -4,47 | 9,57 | 170615DJ |  |  |  |  |
| 40 | -4,50 | 9,50 | 170615DJ |  |  |  |  |
| 40 | -4,64 | 9,53 | 170615DJ |  |  |  |  |
| 40 | -4,36 | 9,61 | 170929DA |  |  |  |  |
| 40 | -4,55 | 9,65 | 170929DA |  |  |  |  |
| 40 | -4,67 | 9,56 | 170929DA |  |  |  |  |
| 40 | -4,49 | 9,08 | 170929DA |  |  |  |  |
| 40 | -4,42 | 9,62 | 170929DB |  |  |  |  |
| 40 | -4,45 | 9,70 | 170929DB |  |  |  |  |
| 40 | -4,71 | 9,58 | 170929DB |  |  |  |  |
| 40 | -4,50 | 9,53 | 170929DB |  |  |  |  |
| 40 | -4,31 | 9,58 | 170929DC |  |  |  |  |
| 40 | -4,44 | 9,63 | 170929DC |  |  |  |  |
| 40 | -4,54 | 9,87 | 170929DC |  |  |  |  |
| 40 | -4,77 | 10,04 | 170929DC |  |  |  |  |
| 40 | -4,27 | 9,55 | 170929DD |  |  |  |  |
| 40 | -4,45 | 9,64 | 170929DD |  |  |  |  |
| 40 | -4,73 | 9,60 | 170929DD |  |  |  |  |
| 40 | -4,63 | 9,51 | 170929DD |  |  |  |  |
| 40 | -4,36 | 9,31 | 170929DE |  |  |  |  |
| 40 | -4,41 | 9,58 | 170929DE |  |  |  |  |
| 40 | -4,69 | 9,57 | 170929DE |  |  |  |  |
| 40 | -4,62 | 9,51 | 170929DE |  |  |  |  |
| 40 | -4,30 | 9,23 | 170929DF |  |  |  |  |
| 40 | -4,36 | 9,56 | 170929DF |  |  |  |  |
| 40 | -4,72 | 9,54 | 170929DF |  |  |  |  |
| 40 | -4,69 | 11,02 | 170929DF |  |  |  |  |
| 40 | -4,41 | 9,53 | 170929DG |  |  |  |  |
| 40 | -4,54 | 8,76 | 170929DG |  |  |  |  |
| 40 | -4,62 | 9,39 | 170929DG |  |  |  |  |
| 40 | -4,51 | 9,28 | 170929DG |  |  |  |  |
| 40 | -4,43 | 10,44 | 170929DH |  |  |  |  |
| 40 | -4,52 | 10,44 | 170929DH |  |  |  |  |
| 40 | -4,52 | 10,04 | 170929DH |  |  |  |  |
| 40 | -4,60 | 9,25 | 170929DH |  |  |  |  |
| 40 | -4,48 | 9,25 | 170929DI |  |  |  |  |
| 40 | -4,60 | 9,53 | 170929DI |  |  |  |  |
| 40 | -4,52 | 9,52 | 170929DI |  |  |  |  |
| 40 | -4,48 | 9,47 | 170929DI |  |  |  |  |
| 40 | -4,39 | 9,62 | 170929DJ |  |  |  |  |
| 40 | -4,32 | 9,73 | 170929DJ |  |  |  |  |
| 40 | -4,59 | 9,45 | 170929DJ |  |  |  |  |
| 40 | -4,68 | 9,27 | 170929DJ |  |  |  |  |
| 40 | -4,61 | 9,32 | 170929DJ |  |  |  |  |
|  |  |  |  |  |  |  |  |
| 41a | 47,16 | 9,52 | 170503DA |  |  |  |  |
| 41a | 47,85 | 9,50 | 170503DA |  |  |  |  |
| 41a | 47,69 | 9,50 | 170503DA |  |  |  |  |
| 41a | 47,53 | 9,67 | 170503DB |  |  |  |  |
| 41a | 47,57 | 9,69 | 170503DB |  |  |  |  |
| 41a | 47,60 | 9,54 | 170503DB |  |  |  |  |
| 41a | 47,34 | 9,65 | 170503DC |  |  |  |  |
| 41a | 47,73 | 9,71 | 170503DC |  |  |  |  |
| 41a | 47,64 | 9,49 | 170503DC |  |  |  |  |
| 41a | 47,63 | 9,76 | 170503DD |  |  |  |  |
| 41a | 47,57 | 9,92 | 170503DD |  |  |  |  |
| 41a | 47,51 | 9,28 | 170503DD |  |  |  |  |
| 41a | 47,54 | 9,78 | 170503DE |  |  |  |  |
| 41a | 47,78 | 9,62 | 170503DE |  |  |  |  |
| 41a | 47,39 | 9,43 | 170503DE |  |  |  |  |
| 41a | 47,44 | 9,60 | 170615DA |  |  |  |  |
| 41a | 47,66 | 9,61 | 170615DA |  |  |  |  |
| 41a | 47,62 | 9,50 | 170615DA |  |  |  |  |
| 41a | 47,63 | 9,61 | 170615DB |  |  |  |  |
| 41a | 47,51 | 9,63 | 170615DB |  |  |  |  |
| 41a | 47,57 | 9,52 | 170615DB |  |  |  |  |
| 41a | 47,59 | 9,73 | 170615DC |  |  |  |  |
| 41a | 47,55 | 10,12 | 170615DC |  |  |  |  |
| 41a | 47,57 | 10,99 | 170615DC |  |  |  |  |
| 41a | 47,51 | 9,52 | 170615DD |  |  |  |  |
| 41a | 47,69 | 9,61 | 170615DD |  |  |  |  |
| 41a | 47,50 | 9,49 | 170615DD |  |  |  |  |
| 41a | 47,46 | 9,67 | 170615DE |  |  |  |  |
| 41a | 47,78 | 9,58 | 170615DE |  |  |  |  |
| 41a | 47,47 | 9,50 | 170615DE |  |  |  |  |
| 41a | 47,42 | 9,57 | 170615DF |  |  |  |  |
| 41a | 47,63 | 9,57 | 170615DF |  |  |  |  |
| 41a | 47,65 | 9,54 | 170615DF |  |  |  |  |
| 41a | 47,38 | 9,42 | 170615DG |  |  |  |  |
| 41a | 47,70 | 9,64 | 170615DG |  |  |  |  |
| 41a | 47,62 | 9,73 | 170615DG |  |  |  |  |
| 41a | 47,49 | 9,58 | 170615DH |  |  |  |  |
| 41a | 47,61 | 9,61 | 170615DH |  |  |  |  |
| 41a | 47,61 | 9,57 | 170615DH |  |  |  |  |
| 41a | 47,45 | 9,69 | 170615DI |  |  |  |  |
| 41a | 47,49 | 9,97 | 170615DI |  |  |  |  |
| 41a | 47,76 | 9,29 | 170615DI |  |  |  |  |
| 41a | 47,53 | 9,63 | 170615DJ |  |  |  |  |
| 41a | 47,65 | 9,51 | 170615DJ |  |  |  |  |
| 41a | 47,52 | 9,57 | 170615DJ |  |  |  |  |
| 41a | 47,57 | 9,75 | 170929DA |  |  |  |  |
| 41a | 47,62 | 9,64 | 170929DA |  |  |  |  |
| 41a | 47,52 | 9,07 | 170929DA |  |  |  |  |
| 41a | 47,49 | 9,80 | 170929DB |  |  |  |  |
| 41a | 47,50 | 9,67 | 170929DB |  |  |  |  |
| 41a | 47,72 | 9,55 | 170929DB |  |  |  |  |
| 41a | 47,86 | 9,19 | 170929DC |  |  |  |  |
| 41a | 47,51 | 10,05 | 170929DC |  |  |  |  |
| 41a | 47,34 | 10,14 | 170929DC |  |  |  |  |
| 41a | 47,75 | 9,78 | 170929DD |  |  |  |  |
| 41a | 47,58 | 9,69 | 170929DD |  |  |  |  |
| 41a | 47,37 | 9,55 | 170929DD |  |  |  |  |
| 41a | 47,77 | 9,63 | 170929DE |  |  |  |  |
| 41a | 47,35 | 9,64 | 170929DE |  |  |  |  |
| 41a | 47,58 | 9,51 | 170929DE |  |  |  |  |
| 41a | 47,70 | 9,56 | 170929DF |  |  |  |  |
| 41a | 47,62 | 9,70 | 170929DF |  |  |  |  |
| 41a | 47,39 | 11,37 | 170929DF |  |  |  |  |
| 41a | 47,60 | 9,53 | 170929DG |  |  |  |  |
| 41a | 47,54 | 9,40 | 170929DG |  |  |  |  |
| 41a | 47,57 | 9,35 | 170929DG |  |  |  |  |
| 41a | 47,61 | 10,45 | 170929DH |  |  |  |  |
| 41a | 47,74 | 10,02 | 170929DH |  |  |  |  |
| 41a | 47,36 | 8,92 | 170929DH |  |  |  |  |
| 41a | 47,40 | 9,67 | 170929DI |  |  |  |  |
| 41a | 47,63 | 9,64 | 170929DI |  |  |  |  |
| 41a | 47,68 | 9,45 | 170929DI |  |  |  |  |
| 41a | 47,59 | 9,38 | 170929DJ |  |  |  |  |
| 41a | 47,51 | 9,56 | 170929DJ |  |  |  |  |
| 41a | 47,55 | 9,37 | 170929DJ |  |  |  |  |
| 41a | 47,63 | 9,39 | 170929DJ |  |  |  |  |
|  |  |  |  |  |  |  |  |
| GA1 QC | -5,28 | 9,42 | 170503DA |  |  |  |  |
| GA1 QC | -5,06 | 9,46 | 170503DA |  |  |  |  |
| GA1 QC | -5,14 | 9,60 | 170503DB |  |  |  |  |
| GA1 QC | -5,16 | 9,54 | 170503DB |  |  |  |  |
| GA1 QC | -5,14 | 9,62 | 170503DC |  |  |  |  |
| GA1 QC | -5,26 | 9,37 | 170503DC |  |  |  |  |
| GA1 QC | -5,42 | 9,68 | 170503DD |  |  |  |  |
| GA1 QC | -5,23 | 9,64 | 170503DD |  |  |  |  |
| GA1 QC | -5,13 | 9,71 | 170503DE |  |  |  |  |
| GA1 QC | -5,28 | 9,56 | 170503DE |  |  |  |  |
| GA1 QC | -5,18 | 9,17 | 170615DA |  |  |  |  |
| GA1 QC | -5,32 | 9,52 | 170615DA |  |  |  |  |
| GA1 QC | -5,36 | 9,48 | 170615DB |  |  |  |  |
| GA1 QC | -5,26 | 7,97 | 170615DB |  |  |  |  |
| GA1 QC | -5,34 | 9,80 | 170615DC |  |  |  |  |
| GA1 QC | -5,26 | 10,33 | 170615DC |  |  |  |  |
| GA1 QC | -5,05 | 9,53 | 170615DD |  |  |  |  |
| GA1 QC | -5,20 | 9,51 | 170615DD |  |  |  |  |
| GA1 QC | -5,12 | 9,69 | 170615DE |  |  |  |  |
| GA1 QC | -5,12 | 9,50 | 170615DE |  |  |  |  |
| GA1 QC | -5,31 | 9,51 | 170615DF |  |  |  |  |
| GA1 QC | -5,32 | 9,37 | 170615DF |  |  |  |  |
| GA1 QC | -5,12 | 9,44 | 170615DG |  |  |  |  |
| GA1 QC | -5,19 | 9,52 | 170615DG |  |  |  |  |
| GA1 QC | -5,20 | 9,48 | 170615DH |  |  |  |  |
| GA1 QC | -5,08 | 9,17 | 170615DH |  |  |  |  |
| GA1 QC | -5,41 | 9,72 | 170615DI |  |  |  |  |
| GA1 QC | -5,29 | 9,66 | 170615DI |  |  |  |  |
| GA1 QC | -5,28 | 9,61 | 170615DJ |  |  |  |  |
| GA1 QC | -5,25 | 9,56 | 170615DJ |  |  |  |  |
| GA1 QC | -5,19 | 9,64 | 170929DA |  |  |  |  |
| GA1 QC | -5,26 | 9,33 | 170929DA |  |  |  |  |
| GA1 QC | -5,16 | 9,67 | 170929DB |  |  |  |  |
| GA1 QC | -5,39 | 9,51 | 170929DB |  |  |  |  |
| GA1 QC | -5,14 | 9,78 | 170929DC |  |  |  |  |
| GA1 QC | -5,26 | 9,11 | 170929DC |  |  |  |  |
| GA1 QC | -5,02 | 7,98 | 170929DD |  |  |  |  |
| GA1 QC | -5,22 | 9,49 | 170929DD |  |  |  |  |
| GA1 QC | -5,21 | 8,31 | 170929DE |  |  |  |  |
| GA1 QC | -5,30 | 9,49 | 170929DE |  |  |  |  |
| GA1 QC | -5,23 | 9,57 | 170929DF |  |  |  |  |
| GA1 QC | -5,19 | 10,26 | 170929DF |  |  |  |  |
| GA1 QC | -5,31 | 9,38 | 170929DG |  |  |  |  |
| GA1 QC | -5,21 | 9,23 | 170929DG |  |  |  |  |
| GA1 QC | -5,17 | 10,23 | 170929DH |  |  |  |  |
| GA1 QC | -5,15 | 9,79 | 170929DH |  |  |  |  |
| GA1 QC | -5,24 | 9,60 | 170929DI |  |  |  |  |
| GA1 QC | -5,19 | 9,55 | 170929DI |  |  |  |  |
| GA1 QC | -5,18 | 9,66 | 170929DJ |  |  |  |  |
| GA1 QC | -5,26 | 9,50 | 170929DJ |  |  |  |  |
| GA1 QC | -5,35 | 9,41 | 170929DJ |  |  |  |  |
|  |  |  |  |  |  |  |  |
| GA2 QC | -3,06 | 9,39 | 170503DA |  |  |  |  |
| GA2 QC | -2,98 | 9,41 | 170503DA |  |  |  |  |
| GA2 QC | -3,14 | 9,67 | 170503DB |  |  |  |  |
| GA2 QC | -3,02 | 9,55 | 170503DB |  |  |  |  |
| GA2 QC | -2,95 | 9,75 | 170503DC |  |  |  |  |
| GA2 QC | -3,01 | 9,50 | 170503DC |  |  |  |  |
| GA2 QC | -3,19 | 9,75 | 170503DD |  |  |  |  |
| GA2 QC | -3,03 | 9,62 | 170503DD |  |  |  |  |
| GA2 QC | -2,95 | 9,70 | 170503DE |  |  |  |  |
| GA2 QC | -3,03 | 9,51 | 170503DE |  |  |  |  |
| GA2 QC | -3,14 | 9,53 | 170615DA |  |  |  |  |
| GA2 QC | -3,17 | 9,50 | 170615DA |  |  |  |  |
| GA2 QC | -3,14 | 9,51 | 170615DB |  |  |  |  |
| GA2 QC | -3,16 | 9,49 | 170615DB |  |  |  |  |
| GA2 QC | -3,08 | 9,83 | 170615DC |  |  |  |  |
| GA2 QC | -3,20 | 10,27 | 170615DC |  |  |  |  |
| GA2 QC | -3,08 | 9,52 | 170615DD |  |  |  |  |
| GA2 QC | -3,00 | 9,50 | 170615DD |  |  |  |  |
| GA2 QC | -2,99 | 9,71 | 170615DE |  |  |  |  |
| GA2 QC | -2,93 | 9,54 | 170615DE |  |  |  |  |
| GA2 QC | -3,22 | 9,46 | 170615DF |  |  |  |  |
| GA2 QC | -3,15 | 9,52 | 170615DF |  |  |  |  |
| GA2 QC | -3,05 | 9,53 | 170615DG |  |  |  |  |
| GA2 QC | -2,94 | 9,60 | 170615DG |  |  |  |  |
| GA2 QC | -3,13 | 9,46 | 170615DH |  |  |  |  |
| GA2 QC | -2,98 | 9,56 | 170615DH |  |  |  |  |
| GA2 QC | -3,18 | 9,75 | 170615DI |  |  |  |  |
| GA2 QC | -3,21 | 9,66 | 170615DI |  |  |  |  |
| GA2 QC | -3,12 | 9,61 | 170615DJ |  |  |  |  |
| GA2 QC | -3,06 | 9,49 | 170615DJ |  |  |  |  |
| GA2 QC | -2,96 | 9,55 | 170929DA |  |  |  |  |
| GA2 QC | -3,10 | 9,37 | 170929DA |  |  |  |  |
| GA2 QC | -3,01 | 9,75 | 170929DB |  |  |  |  |
| GA2 QC | -3,08 | 9,36 | 170929DB |  |  |  |  |
| GA2 QC | -2,92 | 9,73 | 170929DC |  |  |  |  |
| GA2 QC | -3,26 | 10,03 | 170929DC |  |  |  |  |
| GA2 QC | -3,02 | 9,58 | 170929DD |  |  |  |  |
| GA2 QC | -3,17 | 9,52 | 170929DD |  |  |  |  |
| GA2 QC | -3,07 | 9,56 | 170929DE |  |  |  |  |
| GA2 QC | -3,16 | 9,53 | 170929DE |  |  |  |  |
| GA2 QC | -2,95 | 9,57 | 170929DF |  |  |  |  |
| GA2 QC | -3,01 | 10,09 | 170929DF |  |  |  |  |
| GA2 QC | -3,13 | 9,56 | 170929DG |  |  |  |  |
| GA2 QC | -3,12 | 9,52 | 170929DG |  |  |  |  |
| GA2 QC | -2,95 | 10,36 | 170929DH |  |  |  |  |
| GA2 QC | -2,95 | 9,75 | 170929DH |  |  |  |  |
| GA2 QC | -3,08 | 9,56 | 170929DI |  |  |  |  |
| GA2 QC | -3,06 | 9,53 | 170929DI |  |  |  |  |
| GA2 QC | -2,97 | 9,68 | 170929DJ |  |  |  |  |
| GA2 QC | -3,15 | 9,51 | 170929DJ |  |  |  |  |
| GA2 QC | -3,22 | 9,42 | 170929DJ |  |  |  |  |
